# 253. Detecting Vancomycin-Resistant *Enterococcus* (VRE) Gut Colonization in Medical Intensive Care Unit (MICU) Patients: Comparing Culture and Metagenomics

**DOI:** 10.1093/ofid/ofad500.325

**Published:** 2023-11-27

**Authors:** Christine Bassis, Sarah E Sansom, Michael Schoeny, Lahari Thotapalli, Christine Fukuda, Thelma E Dangana, Ali Pirani, Evan Snitkin, Vincent B Young, Mary K Hayden

**Affiliations:** University of Michigan, Ann Arbor, Michigan; Rush University Medical Center, Chicago, IL; Rush University, Chicago, Illinois; Rush University Medical Center, Chicago, IL; Rush University Medical Center, Chicago, IL; Rush University Medical, Chicago, Illinois; University of Michigan, Ann Arbor, Michigan; University of Michigan, Ann Arbor, Michigan; University of Michigan, Ann Arbor, Michigan; Rush University Medical Center, Chicago, IL

## Abstract

**Background:**

Investigation of the colonization dynamics of vancomycin resistant *Enterococcus faecium* (VRE) depends on reliable detection methods. In this study we investigated if metagenomic sequencing enhances our understanding of VRE gut colonization in addition to selective culture.

**Methods:**

We performed metagenomic sequence analysis on 48 stool and rectal swab samples from 15 medical intensive care unit (MICU) patients with dynamic VRE colonization patterns by selective culture. After metagenomic sequences were quality-filtered, trimmed and human sequences were removed, the antimicrobial resistance (AMR) genes were identified using AMR++ v3.0 and the AMR database MEGARes v3.0.

**Results:**

Microbial sequencing depth varied between samples due to low total DNA and varied percentage of human DNA (median: 68.6%; IQR: 55.9-90.5%). We obtained a median of 1.4 million microbial sequence reads/sample (IQR: 0.6 - 6.0 million). Vancomycin resistance gene cluster sequences (Van-type genes) were detected in the metagenomes of 14 samples. There was agreement between VRE-status as by selective culture and detection of Van-type gene sequence in 33/48 samples. 12 samples that were VRE positive by culture did not have Van-type genes detected in the metagenome, possibly due to lower microbial sequencing depth (median: 1 million sequences/sample; IQR: 0.6 - 4.6 million) compared to samples with Van-type genes detected (median: 10.9 million reads/sample; IQR: 1.6 - 29.7 million).

Three VRE culture-negative samples had Van-type genes detected. 2 of these contained the same Van-type genes found in other samples from the same subject, suggesting that they represent actual VRE colonization. In another patient, 2 VRE culture-positive samples collected 15 days apart had the same 11 VanA-type gene sequences, suggesting the same VRE strain was present in both samples.

**Conclusion:**

Metagenomic detection of VRE was influenced by microbial sequencing depth, which is related to total sequencing depth and percentage of host reads. Greater sequencing depth may improve sensitivity of metagenomics to detect resistant organisms. Longitudinal analysis of antimicrobial resistance gene sequences from metagenomes has the potential to provide insight on colonization dynamics.

**Disclosures:**

**Vincent B. Young, MD, PhD**, ASM: Senior Editor for mSphere Journal|Debiopharm: Consultant|mSphere: Senior Editor|Vendanta Biosciences: Consultant

